# Application of Transcranial Sonography for the Assessment of Brain Midline Shift in Patients Presenting With Suspected Intracranial Pathology to the Emergency Department of a Tertiary Care Hospital in Central Gujarat, India

**DOI:** 10.7759/cureus.52561

**Published:** 2024-01-19

**Authors:** Himanshu Gupta, Shreyas K Patel, Atul I Bhoraniya, Nimesh B Malaviya, Rina Parikh, Krunalkumar Pancholi

**Affiliations:** 1 Emergency Medicine, Jaipur National University Institute for Medical Sciences & Research Centre, Jaipur, IND; 2 Emergency Medicine, Parul Institute of Medical Science & Research, Parul University, Vadodara, IND; 3 Emergency Medicine, Gujarat Medical Education & Research Society Medical College, Morbi, IND; 4 Emergency Medicine, Parul Institute of Medical Sciences & Research, Parul University, Vadodara, IND; 5 Emergency Medicine, Sir Sayajirao General (SSG) Hospital & Medical College, Vadodara, IND

**Keywords:** emergency department, emergency medicine, pocus, brain sonography, brain midline shift, transcranial sonography, tcs

## Abstract

Background: A shift in midline brain structure indicates raised intracranial pressure (ICP), thereby a sign of compromised perfusion to brain tissues or a mass effect. Early diagnosis can help in planning timely neurosurgical interventions that could prevent further neuron loss. Also, this may aid in neuroprognostication.

Objectives: The objectives of the study were to find the accuracy of bedside assessment of brain midline shift (MLS) using transcranial sonography (TCS) in comparison to a computed tomography (CT) scan of the brain for patients presenting with suspected intracranial pathology to the emergency department (ED).

Methods: This prospective observational study was carried out for one year in an ED. A total of 124 patients with suspected intracranial pathology were included in the study. Transtemporal scanning along the orbitomeatal line was performed to image the third ventricle. The distance between the third ventricle and the internal side of the temporal bone was measured on both sides as A and B. The MLS was then calculated using the following formula: midline shift = (A-B)/2. The data were entered and analyzed using a Microsoft Excel worksheet (Microsoft Corp., Redmond, WA).

Results: Out of the total 124 patients enrolled in this study, adequate views for 12 patients were not obtained and, hence, they were excluded from the study. The time to perform a TCS assessment of brain MLS was around 22 minutes (range: 15-30 minutes). In our study, out of 112 analyzed patients, 33 (29.5% of our study) had a significant MLS in the brain (defined by an MLS of more than 5 mm) diagnosed by TCS. Analyzing CT brain results revealed that out of the total 112 patients under study, 27 had a significant brain MLS (24.1% of the total population under study) as defined above.

Conclusion: A TCS is a promising alternative to a brain CT in an emergency for brain MLS detection.

## Introduction

A complete neurological examination is not always possible in patients presenting with suspected intracranial pathology. Bedside assessment is limited to the examination of brainstem structures (Glasgow Coma Scale, pupils, Cushing’s reflex, deep tendon reflexes (DTR), and respiratory pattern). Brain midline shift (MLS) is defined as the degree of horizontal shift of midline cerebral structures as seen on axial images and is correlated with clinical state [1]. It is measured in millimeters as the perpendicular distance between midline structures like the septum pellucidum, the third ventricle, or the pineal gland, depending on the reference imaging [2].

Brain MLS is considered an ominous sign because it is commonly associated with a distortion of the brain stem that can cause serious dysfunctions like respiratory failure, coma, and death. It is considered a measure of intracranial pressure (ICP), and the presence of a midline shift is an indicator of raised ICP [3].

In 1977, Becker et al. noted that more than 1 cm of midline shift can lead to a two-fold increase in mortality (53% versus 25%) [4]. A midline shift of 5 mm or more has been shown to predict poor neurological outcomes [5, 6].

Secondary brain injury can be halted by planning early neurosurgical interventions if the development of brain midline shift is diagnosed early. It can also help in neuroprognostication [7-9].

Computed tomography (CT) scans are the main diagnostic modalities for intracranial pathologies in most emergency departments (EDs). Though a gold standard for investigation, a CT scan is not a bedside/readily available tool in the ED. The need for a reliable bedside tool in the ED to identify life-threatening events such as herniation syndrome has been felt for a long time.

The use of bedside transcranial sonography (TCS) has been established for suspected intracranial pathologies such as intracranial space-occupying lesions, stroke syndromes (both ischemic and hemorrhagic), MLS due to traumatic brain injury, and neuro-degenerative movement disorders [10]. Being available at the bedside and bearing low-cost ultrasound gives the medical team an edge with the advantage of repeatability and no radiation exposure. Short study durations save time in emergency settings.

This study was conducted in the emergency department to detect brain MLS in patients presenting with suspected intracranial pathology using TCS as a screening method.

## Materials and methods

This prospective observational study was conducted by an emergency physician in the ED of a tertiary care hospital in central Gujarat, India, spanning over one year from September 2019 to August 2020. Ethical Committee approval was taken from the Institutional Ethics Committee for Human Research (IECHR)of the Post Graduate Research, Medical College & Sir Sayajirao General (SSG) Hospital, Baroda, with No. lECHR-PGR/43-19.

A total of 124 patients with suspected intracranial pathology, whether traumatic or non-traumatic (with signs and symptoms of ICP) who presented in the ED, were included in this study (Table 1).

**Table 1:** Criteria for suspected intracranial pathology ICP: intracranial pressure

Symptoms and signs of raised ICP
Headache, nausea, and vomiting
Cushing’s triad: hypertension, bradycardia, and irregular respiration
Confusion and decreased mental abilities
Diplopia, unequal pupils, and abnormal pupillary light response
Loss of consciousness
Stupor and coma

Out of the total 124 patients enrolled in this study, in 12 patients, adequate views were not obtained by the emergency physician and, hence, were excluded from the study.

Description of TCS in MLS assessment

When a patient with suspected intracranial pathology presented to the ED, the patient was first resuscitated, and simultaneously, a primary assessment and emergency clinical examination, including point-of-care ultrasound (POCUS), were done. The description of the TCS method is given in Table 2.

**Table 2:** Description of the transcranial sonography (TCS) process

	Description of the method
Patient position	The patient has to be kept in a supine position with the examiner at the head end of the patient.
Machine and probe	A low-frequency (2–5 MHz) phased array probe of the MyLab20 Esaote USG machine was used with cephalic mode settings.
Probe positioning	A transtemporal window can be obtained by positioning the probe in the temporal zone close to the pterion along the orbitomeatal line. The pterion is the thinnest portion of the temporal bone, located cephalad to the zygomatic arch and anterior to the ear.
Probe marker	Probe index mark orientation towards the patient’s anterior/front

Midline shift assessment

**Figure 1:**
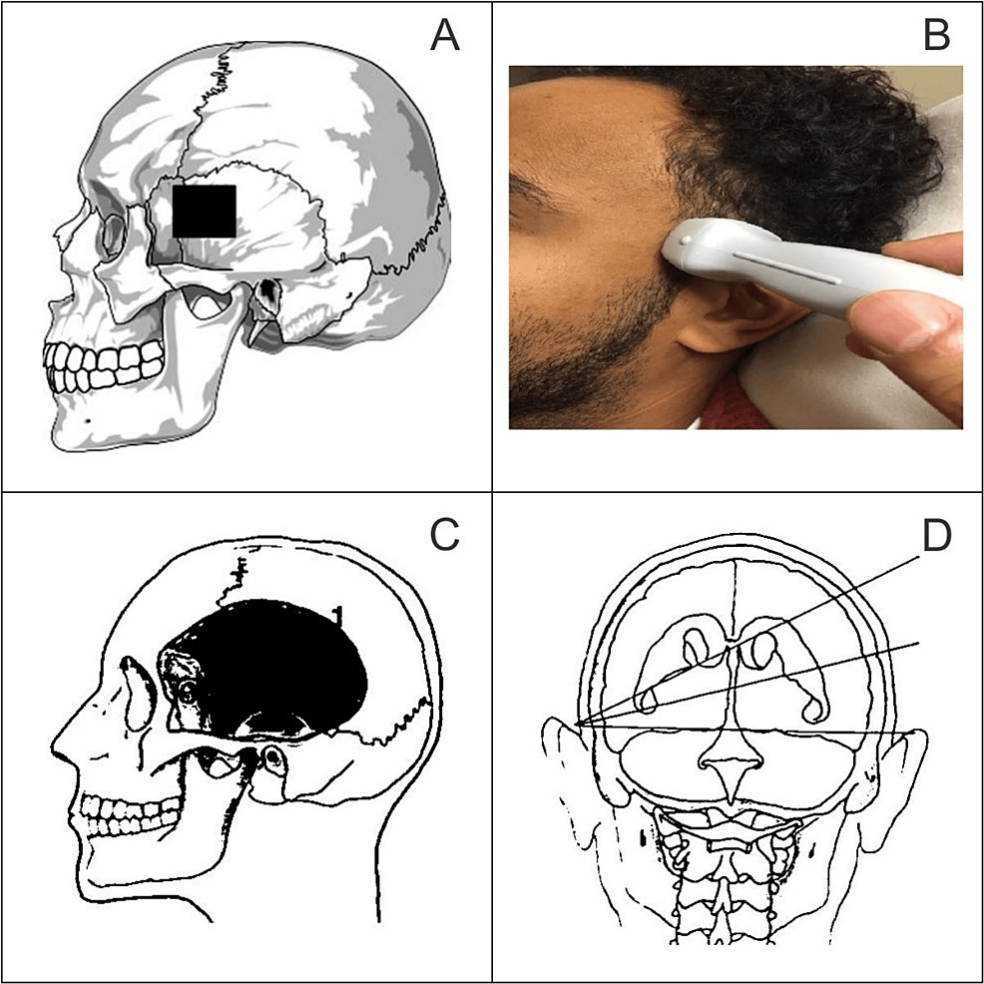
Transcranial sonography location A. location of the pterion, B. method for the acquisition of transcranial Doppler; the marker of the probe should be towards the patient’s anterior side. C. transcranial sonographic scanning planes; D. transcranial sonographic scanning area

The examination starts with scanning in the axial plane parallel to the orbitomeatal line at midbrain level, as shown in Figure 1.

**Figure 2:**
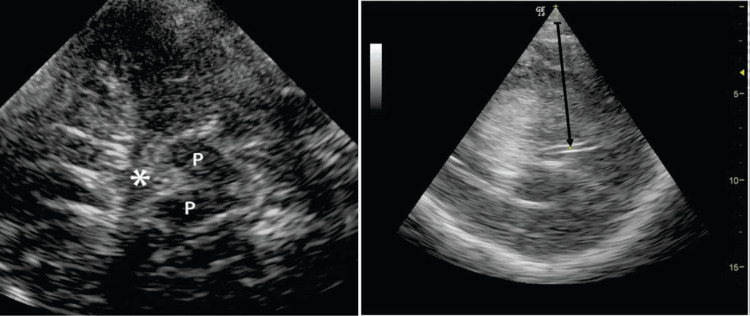
Transtemporal gray-scale image showing trans-temporal visualization of the third ventricle of the cerebral peduncles (P) with the echogenic structures with an arrow pointing towards the center. The basilar cistern (*) is located just anteriorly.

The first step is to detect an anechogenic or hypoechoic structure shaped like a butterfly, which is the mesencephalic brainstem, which is surrounded by the echogenic structure, which is the basal cisterns shown in Figure 2.

**Figure 3:**
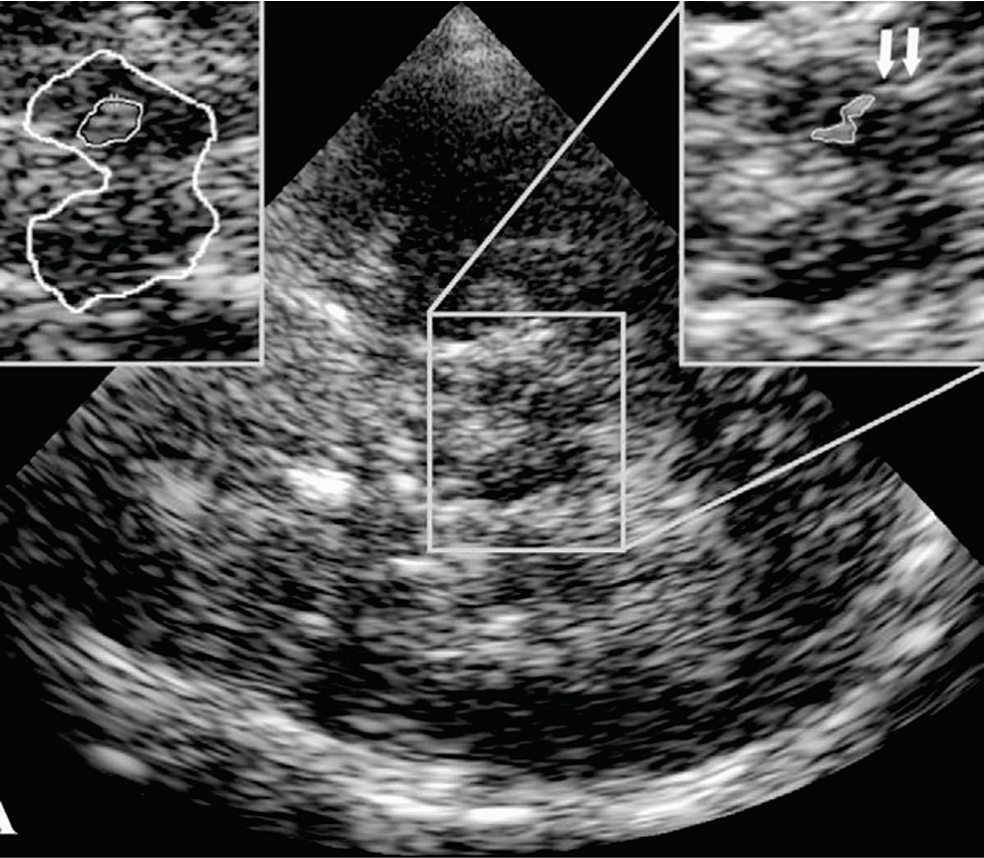
Illustration of transcranial sonography of the axial image of the brain at midbrain level The brainstem, shaped like a butterfly, is displayed in the center, surrounded by the basal cisterns, which are echogenic structures. The zoomed image is shown in a rectangle, demonstrating the midbrain in the right upper corner. Note the typical imaging artifact from the basal cistern (arrows).

**Figure 4:**
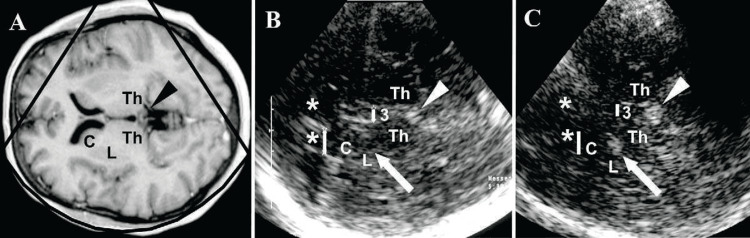
Transcranial sonography (TCS) of the brain at the level of the thalamus. (A) shows an MRI scan, while (B) and (C) show corresponding TCS images. Arrows in (B) and (C) correspond to lenticular nuclei. C: caudate nucleus; L: lenticular nucleus; Th: thalamus; 3: third ventricle (bar is indicating width); *lateral ventricle’s frontal horn (bar is indicating contralateral frontal horn’s width); pineal gland shown as a triangle.

To proceed further, the probe is tilted 10° in an upward direction to display the plane at the thalamus level. Here, we can see a highly echogenic double-line image because of the hyperechogenic ependyma, which is the third ventricle. The other important structures are the hypoechogenic oval thalami and the calcified and echogenic pineal gland. At this level, the transverse diameter of the third ventricle can be measured, as shown in Figures 3, 4.

Calculating the MLS

Seidel et al. also suggested that the midline should be counted at the level of the third ventricle [11]. The distance between the third ventricle and the internal side of the temporal bone (A) needs to be measured. The same calculation can be repeated for the contralateral side (B).

The MLS of the third ventricle is then estimated according to the formula: Midline shift = (A - B)/2.

**Figure 5:**
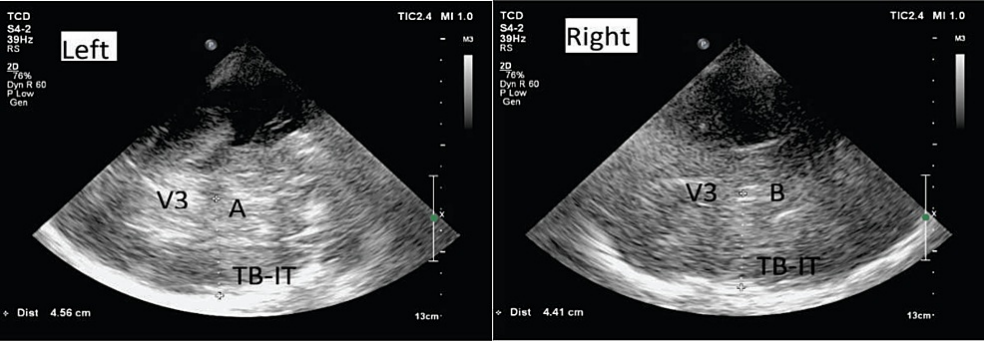
Calculating the midline shift (i) The distance from the third ventricle (V3) to the inner table of the temporal bone (TB-IT) on the left is calculated as A; (ii) the distance from the third ventricle to the inner table of the temporal bone on the right is calculated as B.

By convention, “A” represents the measure taken on the left side and “B” on the right side. The resulting values were thus positive if the mass effect was right-sided and negative if the mass effect was left-sided, as shown in Figure 5.

**Figure 6:**
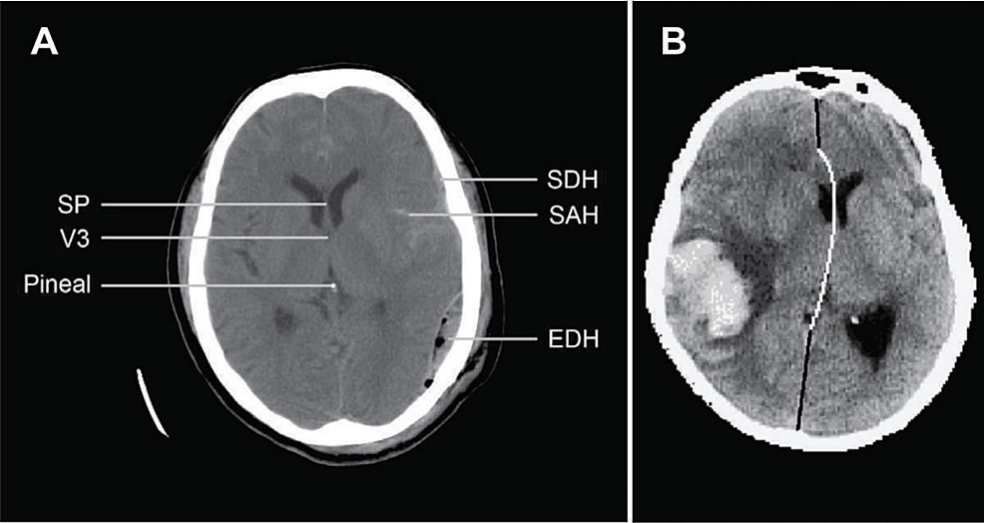
(A) is a CT image of a traumatic brain injury with various anatomical landmarks, which is used to measure the midline shift (MLS) along with different intracranial hemorrhages. (B) is suggestive of MLS due to intracerebral hematoma (ICH) that compresses the brain. SP: septum pellucidum; V3: third ventricle (only the most rostral part shown); SDH: subdural hematoma; SAH: subarachnoid hemorrhage; EDH: epidural hematoma

**Figure 7:**
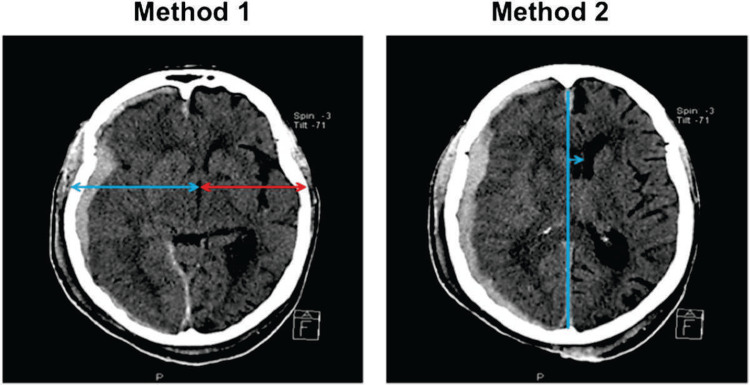
Method 1 shows a method of MLS measurement with a CT scan through the third ventricle, the same as TCS; Method 2 shows another method of measurement between the ideal median line and the septum pellucidum. MLS: midline shift; TCS: transcranial sonography

The MLS thus calculated is compared with the measurements of MLS from the head CT performed by the radiologists, as shown in Figures 6, 7.

## Results

In this study, among 112 patients, 61 were males and 51 were females. The majority (54.5%) were males. Among females, two patients were postpartum, comprising about 4% of the total female population. Age distribution in this study, as summarized in Table 3, shows that the majority of patients (around 65.2%) were from the age group of 19-59 years of age.

**Table 3:** Age-wise demographic data

Age (years)	Males	% among total males	Females	% among total females	Total	% among total patients
Pediatric: 0-12	6	9.8	5	9.8	11	9.8
Adolescents: 13-18	2	3.3	3	5.9	5	4.5
Adults: 19-59	39	63.9	34	66.7	73	65.2
Elderly: ≥60	14	23	9	17.6	23	20.5

The most common presenting symptom was loss of consciousness followed by head injury, vomiting, and headache (Table 4).

**Table 4:** Patients' presenting history

Presenting history	Males	% among total males	Females	% among total females	Total	% among total patients
Head injury	32	52.5	28	45.9	60	53.6
Hemiplegia	22	36.1	18	35.3	40	35.7
Loss of consciousness	49	80.3	34	66.7	83	74.1
Vomiting	34	55.7	25	49	59	52.7
ENT bleeding	10	16.4	5	9.8	15	13.4
Seizure	18	29.5	12	23.5	30	26.8
Headache	37	60.7	21	41.1	58	51.8

A total of 60 patients were presented with traumatic brain injuries, while 52 patients had non-traumatic brain injuries (Table 5).

**Table 5:** Traumatic vs. non-traumatic brain injury

Brain injury	Males	% among total males	Females	% among total females	Total	% among total patients
Traumatic	32	52.5	28	54.9	60	53.6
Non-traumatic	29	47.5	23	45.1	52	46.4

Most patients were responding to voice, followed by pain (Table 6).

**Table 6:** Level of consciousness: AVPU scale and GCS AVPU: alert, voice, pain, unresponsive; GCS: Glasgow coma scale

AVPU/GCS score	Males	% among total males	Females	% among total females	Total	% among total patients
Alert (AVPU score)	3	4.9	9	17.6	12	10.7
Responds to voice (AVPU score)	34	55.8	22	43.1	56	50
Responds to pain (AVPU score)	22	36.1	20	39.3	42	37.5
Unresponsive (AVPU score)	2	3.2	0	0	2	1.8
13-15 GCS score	16	26.2	24	47.1	40	35.7
9-12 GC GCS score	40	65.6	25	49	65	58
< 8 GC GCS score	5	8.2	2	3.9	7	6.3

A focal neurological deficit was found in 82 of 112 patients. The most common deficit was abnormal deep tendon reflexes, followed by hemiplegia (Table 7).

**Table 7:** Focal neurological deficit DTR: deep tendon reflex

Focal neurological deficit	Males	% among total males	Females	% among total females	Total	% among total patients
Hemiplegia	16	26.2	17	33.3	33	29.5
Seizure	9	14.8	7	13.7	16	14.3
Abnormal DTR	25	41	9	17.6	34	30.4
Hemiparesis	4	6.6	1	2	5	4.5
None	10	16.4	20	39.2	30	26.8

Analysis of pupillary asymmetry and light reaction revealed that most patients had bilaterally symmetrical pupils and a normal reaction to light. Of 112 patients, 39 had abnormal pupils in the form of asymmetry and reactivity to light (Table 8).

**Table 8:** Pupillary size and light reaction

Pupils	Males	% among total males	Females	% among total females	Total	% among total patients
Abnormal size or reaction	24	39.3	15	29.4	39	34.8
Bilaterally normal size and reaction	37	60.7	36	70.6	73	65.2

Most patients presented with a normal heart rate, while bradycardia was seen in four patients, and tachycardia was noted in 35 of the total patients (Table 9).

**Table 9:** Vital parameters: heart rate and systolic blood pressure (mm of Hg)

Vital Parameters	Males	% among total males	Females	% among total females	Total	% among total patients
Bradycardia (<60 bpm)	2	3.3	2	3.9	4	3.5
Normal heart rate (60-100 bpm)	37	60.7	36	70.6	73	65.2
Tachycardia (>100 bpm)	22	36.1	13	25.5	35	31.3
≥140 systolic blood pressure	39	63.9	27	52.9	66	58.9
<140 systolic blood pressure	22	36.1	24	47.1	46	41.1

As defined, a significant brain MLS corresponds to a value ≥5mm. In this study, a total of 33 patients (20 males and 13 females) were found to have significant brain MLS with the help of TCS. This constituted around 29.5% of the population under study. The remaining 75.5% of patients (40 males and 39 females) did not have a significant brain MLS on TCS (Table 10).

**Table 10:** Brain midline shift: POCUS (TCS) and CT-brain POCUS: point-of-care ultrasound; TCS: transcranial sonography

Brain midline shift	Males	% among total males	Females	% among total females	Total	% among total patients
≥5 mm in TCS	21	34.4	12	23.5	33	29.5
<5 mm in TCS	40	65.6	39	76.5	79	70.5
≥5 mm in CT brain	16	26.2	11	21.6	27	24.1
<5 mm in CT brain	45	73.8	40	78.4	85	75.9

The CT scan results identified a significant brain MLS in 27 patients (16 males and 11 females), amounting to around 24.1% of the population under study. There were 85 patients (40 males and 40 females) with no significant brain MLS on the brain CT scan.

**Figure 8:**
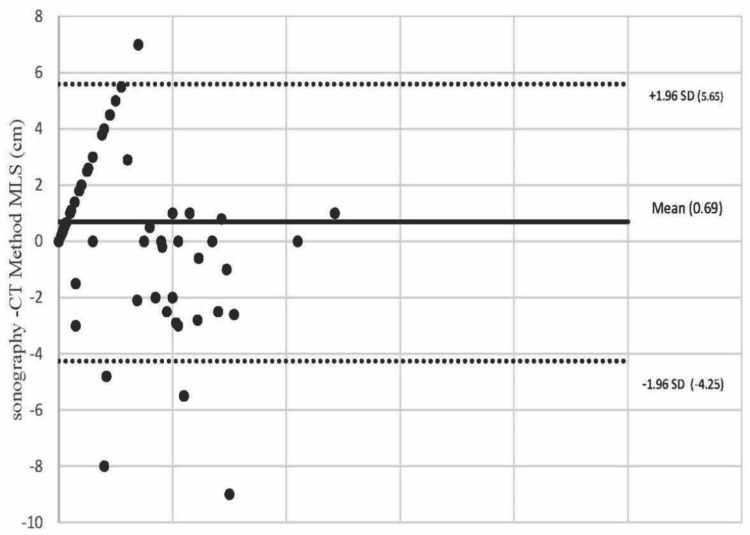
Bland and Altman plot shows the limits of agreement for MLS measurements with the TCS and CT scan methods. MLS: midline shift; TCS: transcranial sonography

The Bland and Altman plot shows the limits of agreement for MLS measurements with the TCS and CT scan methods. The reflecting bias of 0.69 cm and -4.25 to 5.65 cm are the limits of agreement. Only five measurements (4.5% of all) fall outside the limit (Figure 8).

Reliability indices were calculated using standard formulas. The sensitivity calculated came to be 88.89%. Specificity was 89.41%. The positive predictive value came to 72.73%. The negative predictive value was calculated at 96.20% (Table 11).

**Table 11:** Reliability indices TCS: transcranial sonography

Significant brain midline shift: TCS (≥5 mm)	Significant brain midline shift: CT brain (≥5 mm)	
	Positive	Negative
Positive	24	9
Negative	3	76
True positives	24	
False positives	9	
True negatives	76	
False negatives	3	
Sensitivity	88.89 %	
Specificity	89.41 %	
Positive predictive value	72.73 %	
Negative predictive value	96.20%	

**Figure 9:**
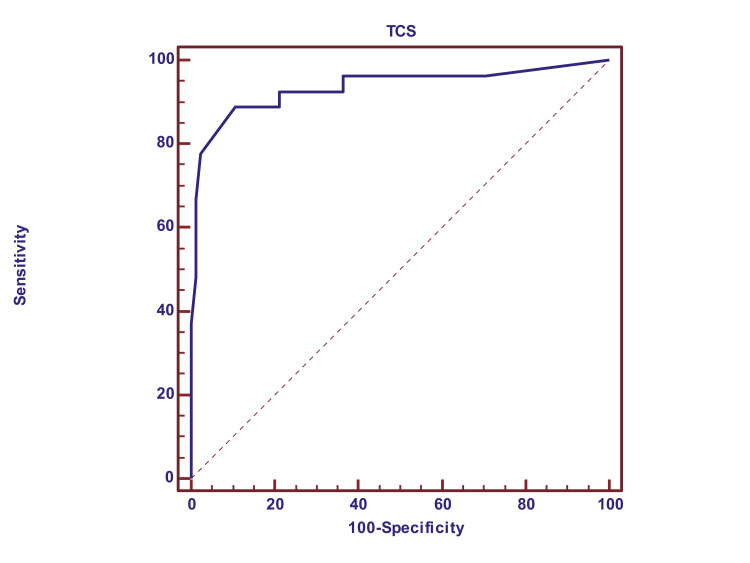
ROC curve for TCS in comparison with CT brain ROC: receiver operating characteristic; TCS: transcranial sonography

Pearson's correlation coefficient is suggestive (R = 0.775, P<0.0001) and highly significant at a 95% confidence interval (0.69 to 0.84). The receiver operating characteristic (ROC) curve is suggestive of 94% of the area under the curve, which is a highly significant co-relation of TCS with CT brain in the measurement of brain midline shift (Figure 9).

## Discussion

Despite the different functionality of both hemispheres of the brain, morphologically, both are identical. Some minor structural differences are insignificant in clinical neuroradiology [12]. Detection of a shift in the calcified pineal gland on plain X-rays in earlier days, followed by pneumoencephalography, were some of the earlier methods to confirm clinical suspicion of raised ICPs and mass effects [12].

A brain MLS, if present, indicates a raised ICP, suggesting a poorer prognosis [13, 14]. Early identification of raised ICP as indicated by significant brain MLS has always been a great concern in emergency departments to institute early measures to maintain ICP to prevent secondary brain injury. Clinical evaluation to reveal the raised ICP in critically ill patients is usually not specific and time-consuming. Physical findings can easily overlap and are not consistently found in all patients. This has led to a huge dependence on CT scans, hindering early resuscitation. Bedside ultrasound can overcome these problems and may help in developing an early resuscitation protocol for patients with raised ICP.

Identification of brain MLS using TCS can aid in the rapid assessment of patients presenting with suspected intracranial pathology to the ED. A TCS can be used for a goal-directed ultrasound assessment of brain MLS, taking the center of the third ventricle as a reference [15].

In this study, a total of 124 patients with clinical features suggesting intracranial pathology were enrolled during the period of one year (from September 2019 to August 2020). Out of these, 54.5% were males and 45.5% were females. Analyzing the studies carried out, the present study shows a male predominance. The ongoing COVID-19 pandemic and the lockdown duration falling within the study duration led to a fall in the overall caseload of the hospital. This impacted the study in terms of the reduced number of cases over the study duration.

Out of the total 124 patients enrolled in this study, adequate views were not obtained for 12 patients, and hence they were excluded from the study. This is consistent with the study by Seidel et al. that states that, due to thicker cranial vaults causing higher bone attenuation, 5%-20% of patients have non-interpretable TCS windows and images [16].

In this study, TCS was carried out with simultaneous resuscitation of the patients. The mean time of sonographic diagnosis from the arrival of the patient in the ED was about 22 minutes (in the range of 15 to 30 minutes).

The present study analyzes both traumatic and non-traumatic brain injury patients. Of the total 112 patients, 60 (53.6% of the total population studied) had a history of head injury (traumatic brain injury), and a total of 52 patients had a non-traumatic brain injury (46.4% of the total population under study). Reliability indices are calculated using standard formulas. The sensitivity calculated came to be 88.89%. Specificity was 89.41%. The present study compares well with the study by Motuel et al. [17].

This study suggests that with the help of TCS, MLS can be diagnosed with reasonable accuracy, particularly in patients with various intracranial abnormalities. Hence, it is useful in early diagnosing and treating significant intracranial pathologies and their mass effects.

The TCS should be used in the ED as a screening method to detect brain MLS in patients presenting with suspected intracranial pathology. However, we have to be aware of the fact that TCS examinations are operator-dependent, and interpretation highly depends on the skills and experience of the operator. The limitation of our study is the small sample size; we need to perform it on a larger scale to extrapolate it. In our study, we have collected data on MLS >5 mm or <5 mm, we have not emphasized the reason for MLS, correlation with severity of shift, clinical presentation, and trauma as well as the accuracy of diagnosis made by TCS. Further study is required to get more information on it.

## Conclusions

In patients with clinical features suggestive of intracranial pathology, this study is a great aid in detecting brain MLS using TCS in critically ill patients presenting to the ED. The TCS examination is non-invasive with less than minimal risk to the patient. It is a bedside procedure and is particularly useful as a screening test and to triage patients to facilitate early diagnosis and resuscitation for patients with significant intracranial mass effects and/or hemodynamically unstable patients. This study can surely guide the clinician to perform a timely, life-saving resuscitative intervention.
